# The need for supportive mental wellbeing interventions in bladder cancer patients: A systematic review of the literature

**DOI:** 10.1371/journal.pone.0243136

**Published:** 2021-01-28

**Authors:** Agustina Bessa, Elke Rammant, Deborah Enting, Richard T. Bryan, Muhammad Shamim Khan, Sachin Malde, Rajesh Nair, Ramesh Thurairaja, Fidelma Cahill, Suzanne Amery, Sue Smith, Kamran Ahmed, Beth Russell, Charlotte Moss, Kathryn Chatterton, Christel Häggström, Mieke Van Hemelrijck

**Affiliations:** 1 School of Cancer and Pharmaceutical Studies, Translational Oncology & Urology Research (TOUR), King’s College London, London, United Kingdom; 2 Department of Radiation Oncology, Ghent University, Ghent, Belgium; 3 Dept. of Oncology, Guy’s and St Thomas’ NHS Foundation Trust, London, United Kingdom; 4 Bladder Cancer Research Centre, Institute of Cancer & Genomic Sciences, University of Birmingham, Birmingham, United Kingdom; 5 Dept. of Psychology, Guy’s and St Thomas’ NHS Foundation Trust, London, United Kingdom; 6 Department of Surgical Sciences, Uppsala University, Uppsala, Sweden; 7 Department of Biobank Research, Umeå University, Umeå, Sweden; University of Maryland School of Medicine, UNITED STATES

## Abstract

**Objectives:**

There is an increased awareness of the effect of a bladder cancer diagnosis and its treatments on the mental wellbeing of patients. However, few studies have evaluated the efficacy, feasibility and acceptability of interventions to improve this mental wellbeing. This systematic review is the first phase of the Medical Research Council Framework for developing complex interventions and provides an overview of the published mental wellbeing interventions that could be used to design an intervention specific for BC patients.

**Methods:**

This review was conducted in accordance with the PRISMA guidelines in January 2019 and studies were identified by conducting searches for Medline, the Cochrane Central Register of Controlled Trials and Ovid Gateway. All included studies met the following criteria: mental wellbeing interventions of adults with medically confirmed diagnosis of any type of urological cancer, reported outcomes for specific HRQoL domains including psychological factors. The quality of evidence was assessed according to Down and Black 27-item checklist.

**Results:**

A total of 15,094 records were collected from the literature search and 10 studies matched the inclusion and exclusion criteria. Of these, nine interventions were for patients with prostate cancer and one for patients with kidney cancer. No studies were found for other urological cancers. Depression was the most commonly reported endpoint measured. Of the included studies with positive efficacy, three were group interventions and two were couple interventions. In the group interventions, all showed a reduction in depressive symptoms and in the couple interventions, there was a reduction in depressive symptoms and a favourable relationship cohesion. The couple interventions were the most feasible and acceptable, but further research was required for most of the studies.

**Conclusion:**

While awareness of the importance of mental wellbeing in bladder cancer patients is growing, this systematic literature review highlights the gap of feasible and acceptable interventions for this patient population.

## 1. Introduction

Bladder cancer (BC) is the 9th most common malignancy worldwide [1] and it is well known that these patients are subjected to significant treatment burdens that are emotionally and psychologically taxing [2]. Many treatment options result in significant decreases in health-related quality of life (HRQoL), which may increase the risk of mental wellbeing issues such as depression, anxiety and stress. The World Health Organization (WHO) states that mental wellbeing includes cognitive, emotional and behavioural responses at a personal level. It should be interpreted in the sociocultural context of the individual [3]. Mental wellbeing complications are apparent in bladder cancer patients as they often have to learn how to cope with their ‘post-surgery body’, changing sexuality and incontinence—all events which can be distressful to the patient [4].

Moreover, it has been observed that patients with BC are at increased risk of suicide compared with the general population. For example, a study based on the Survey, Epidemiology, and End Results (SEER) database assessed suicide rates in patients diagnosed with BC from 1988 to 2010 and identified a standard mortality ratio of 2.71 (as compared to the general population)–with an even higher incidence of suicide for those who underwent radical cystectomy (3.54) [5]. This highlights the importance of filling the unmet supportive care needs among those patients, particularly in terms of psychological and psychosocial support.

With the goal to develop a mental wellbeing intervention to support BC patient needs, we aimed to assess the existing evidence through literature review as per the recommendations of the Medical Research Council (MRC) Framework for developing complex interventions. The MRC Framework recommends that the development phase of such a complex intervention should start with the assessment of the existing evidence through literature review [6]. However, whilst there is an increase in systematic reviews about the effect of a BC diagnosis and its treatments on the mental wellbeing of patients, few studies have evaluated interventions to specifically improve the mental wellbeing of these patients. Therefore, this systematic review aims to report on published mental wellbeing interventions for all urological cancer patients as they all share similar ‘post-surgery body’, changing sexuality and incontinence challenges. This is the first step to understand the efficacy, feasibility and acceptability of mental wellbeing interventions for BC patients.

## 2. Methods

This review was conducted in accordance with the Preferred Reporting Items for Systematic Reviews and Meta-analyses (PRISMA) guidelines. A detailed overview of the protocol is provided in S1 Appendix.

### 2.1. Search strategy and inclusion/exclusion criteria

An assessment of the literature was performed according to PRISMA guidelines in January 2019. Studies were identified by conducting searches for Medline (using the PubMed interface), the Cochrane Central Register of Controlled Trials (CENTRAL) and Ovid Gateway (Embase and Ovid) using a list of defined search terms (see S2 Appendix). To be included in the analysis, the studies must have met the following criteria: mental wellbeing interventions of adults with medically confirmed diagnosis of any type of urological cancer, reported outcomes for specific HRQoL domains including psychological factors such as anxiety, depression, stress and self-esteem, and being published in the last 10 years. Irrelevant studies based on title and abstracts were independently excluded. QOL articles evaluating physical or functional outcomes (i.e., sexual or urinary function) without measurements of mental health were also excluded.

### 2.2. Data collection and analysis

Initially, the titles of the studies were screened to identify the relevant studies. The abstracts and subsequently full texts were then read to identify those which met the inclusion criteria. Information on patient characteristics, number of study participants and type of intervention, as well as efficacy, feasibility, and acceptability were extracted from each study. The search was conducted by two independent reviewers.

### 2.3. Quality assessment of studies

The Downs and Black 27-item checklist was used to assess the quality. Quality of evidence according to Down and Black 27-item checklist is summarised in S3 Appendix. A full description of the Down and Black 27-item system is described elsewhere [7].

### 2.4. Patient and public involvement

No patient involved.

## 3. Evidence synthesis

**Fig 1:**
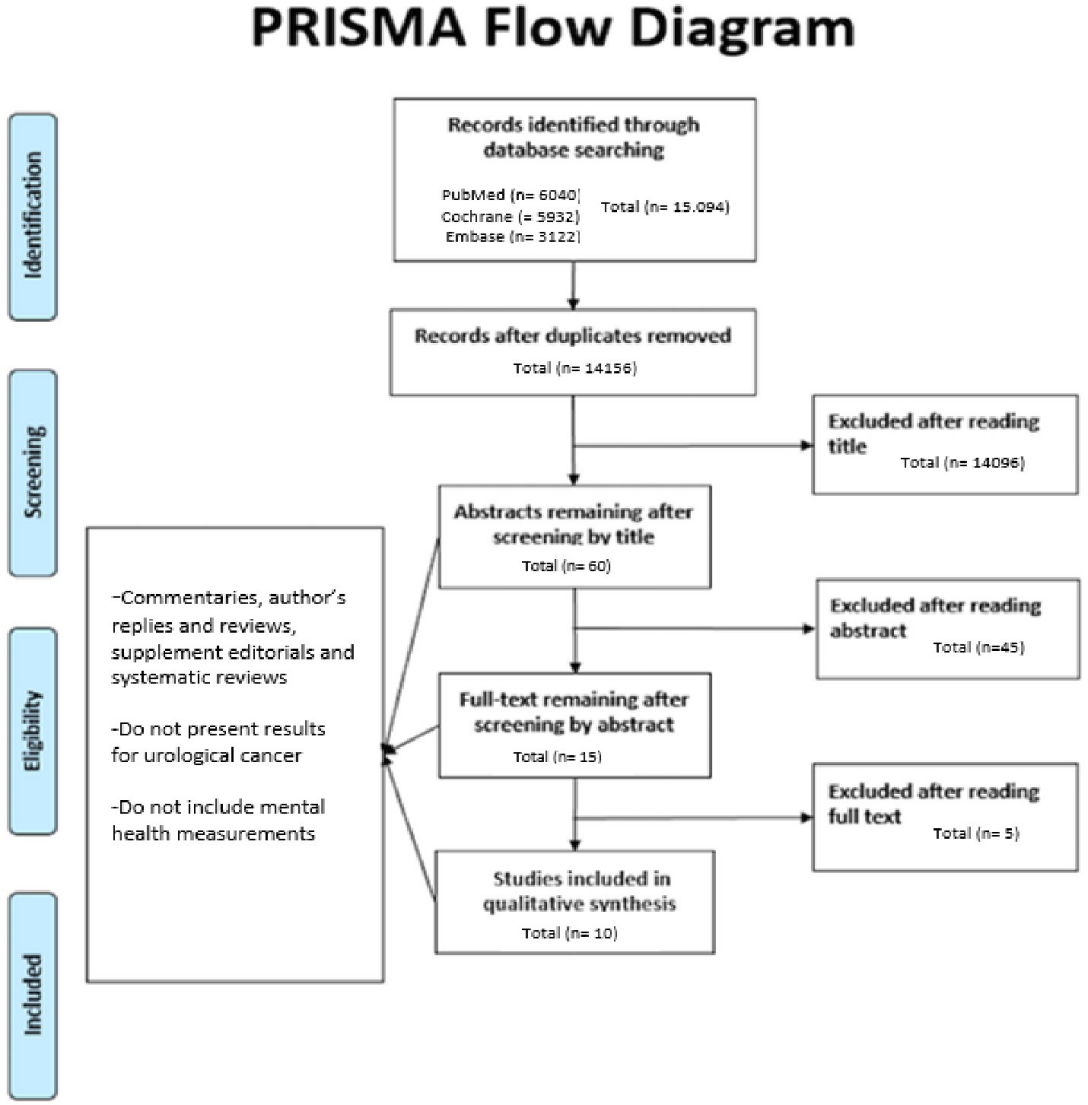
PRISMA Flow diagram for selection of studies in systematic review.

The selection process for records to be included in the review was carried out according to PRISMA protocol, and this is demonstrated in a PRISMA flowchart in Fig 1. A total of 15,094 records were collected from the literature search and 938 duplicates were removed. All titles were initially screened and 60 remained for abstracts screening. Of those, 15 remained for full text analysis. After the full text was read, 10 studies matched the inclusion and exclusion criteria and were included in this systematic review.

Quality of evidence according to Down and Black 27-item checklist is summarised in (Table 2 in S3 Appendix). The checklist provides an overall score for study quality and a profile of scores not only for the quality of reporting, internal validity and power, but also for external validity. Owing to significant heterogeneity of study design and outcomes assessed, the overall quality of evidence was 16/28 suggesting that the studies included address important questions, are well designed, and add support for other findings, but did not contribute substantially with new knowledge.

Of the 10 included interventions, nine interventions were for patients with prostate cancer and one for patients with kidney cancer. The intervention in kidney cancer was conducted in the USA. Among the interventions conducted for patients with prostate cancer, four were in Australia, two were in USA, one in Malaysia, one in UK and one in Sweden. Four studies were conducted as RCTs [8–11]. Three interventions were group consultations [8,9,12], two were individual studies [13,14], two were couple therapy [10,11], two were technology based (via phone or online support) [12,15] and one was a relaxation training [16].

The outcomes measured in each study are summarised in Table 1, with depression being the most commonly reported endpoint. Social, relationship and/or familial wellbeing were also well distributed through the majority of the studies. Depression was assessed using different tools: Depressive Symptoms were assessed using the Center for Epidemiologic Studies Depression Scale (CES-D) in two studies [6, 8], with the Hospital Anxiety and Depression Scale (HADS) in three studies [17,18,20] and using Patient Reported Outcomes Measurement Information System (PROMIS0 depression itembank CAT in one study [11] and self-administered Depression Anxiety Stress Scale (DASS-21) in one study [16].

**Table 1:** Descriptive table of the studies included in the systematic review.

Study ID	Reference	Intervention	Study design	Study population (eligibility criteria)	Sample size (intervention/comparison)	Outcome(s)	Efficacy results	Feasibility and Acceptability of intervention
**Kidney cancer**								
1	[13]	Expressive writing targeting QoL	RCT	Newly diagnosed patients with stage I–IV renal cell carcinoma with no history of psychotherapy	Expressive writing (n = 139) / Neutral writing (n = 138)	Cancer-related symptoms, Depressive Symptoms, Fatigue, Sleep Disturbance, Social Support, Intrusive thoughts and avoidance behaviours	Only positive effect of intervention on cancer-related outcomes for those who reported high depressive symptoms at baseline. No other effects observed.	Intervention may improve QoL if patients also have social support available—for those with no social support the intervention may even be contraindicated
**Prostate cancer**								
2	[6]	Group consultation intervention	RCT	Prostate cancer patients receiving curative intent radiotherapy	Group consultation intervention (n = 165) / Individual consultations (n = 166)	Intervention fidelity, Questionnaire compliance, Outcome analyses, Depressive symptoms, Anxious symptoms, Global distress, Prostate cancer-specific HRQoL, Unmet supportive care needs, Cancer treatment-related concerns	Slight reduction in depressive symptoms in the intervention group between baseline and end of radiotherapy while an increase was observed in the comparison group. Reduction in anxious symptoms for both groups at follow-up assessments from baseline levels. Mean changes between analysis were negligible.	HRQoL and unmet needs advantages were not observed. Findings suggest that group consultations provide an efficient and effective means of delivering patient education.
3	[9]	Mindfulness-Based Cognitive Therapy		Men with advanced prostate cancer (proven metastatic and/or castration-resistant biochemical progression)	Mindfulness cognitive-based therapy (n = 94) / Patient education material (n = 95)	Psychological or cancer-specific distress and quality of life	No significant interactions between study condition and time were found in any outcome studied.	More rigorous evaluations are needed before mindfulness-based approaches can be applied.
4	[8]	Cognitive existential couple therapy (CECT) in men and partners	RCT	Men and partners facing localised prostate cancer	Cognitive existential couple therapy (n = 30) / Usual medical care and information booklet (n = 32)	Relationship functioning, coping, cancer distress, and general mental health	Patients in the intervention grouped demonstrated favourable coping resources and greater relationship cohesion. Younger CECT patients also demonstrated less avoidance.	Intervention provide lower cancer-distress for partners and generated some enduring benefits in relational function. However, CECT should target younger couples.
							Partners in the intervention group demonstrated better psychological well-being, greater use of problem-focused coping strategies and relationship cohesion.	
5	[12]	Technology-assisted group-based psychosocial intervention		Advanced prostate cancer at initial diagnosis	Cognitive-behavioural stress management (n = 37) / Attention-Control Health Promotion Condition (n = 37)	Feasibility, acceptability, Cancer-Related Distress, Depressive Symptoms, Health-Related Quality of Life, Stress Management, Skills Self-Efficacy	The intervention group reported fewer depressive symptoms than the control group. Additionally, participants in the group condition evidenced better outcomes than participants in the control group in intrusive thoughts, emotional well-being.	The findings suggest that technology-assisted interventions can be efficacious and accepted by oncology patients.
6	[11]	Couple-based psychosexual Support		Prostate cancer patients who underwent a radical prostatectomy	Couple-based psychosexual support (n = 21) / Usual follow-up hospital appointment (n = 22)	Acceptability and feasibility, “sexual bother” subdomain, hospital anxiety and depression and family functioning	There was a significant difference on sexual bother for men randomized to the psychosexual intervention group compared with men in the usual-care group; this difference was not maintained at second follow-up. No differences between the intervention and control groups were observed for anxiety, depression, emotional functioning, and relational functioning.	These findings indicate the value of combining a family-systems approach with elements of sex therapy to address broader relational issues that affect sexual function.
7	[26]	Telephone-delivered psychosocial interventions		Prostate cancer patients currently undergoing or had completed treatment within the past 6 months	Interpersonal counseling intervention (n = 35) / Health education attention condition (n = 36)	Psychological well-being (depression, positive and negative affect, stress), physical well-being (fatigue), social well-being, spiritual well-being	The survivors in the TIP-C condition did not exhibit any significant changes on any of the QOL outcomes over time. In contrast, the men in the HEAC condition showed significant changes all in the direction of improved QoL. There was significant improvement in psychological well-being, perceived stress, physical well-being, fatigue, social well-being, increased social support from family members, and spiritual well-being	The psychosocial interventions in this study were effective in improving the multiple dimensions of QoL for men with prostate cancer and their partners. Both the survivor and their intimate partner or family member benefitted from the interventions. These results should be interpreted with caution given the small sample where QoL was relatively high.
8	[14]	Tele-based psycho-educational intervention		Men after diagnosis and before prostate cancer treatment	Telephone delivered counselling session (n = 372) / standard medical management (n = 368)	Cancer-specific psychological distress, decision related distress, Cognitive judgmental adjustment, subjective well-being, Health-related quality of life, treatment side effects	None of the primary outcomes showed an effect of the trial alone. There were slight differences in subgroups analysis.	The study failed to find unconditioned effects for the intervention and propose that the answer to this may lie in interindividual heterogeneity.
9	[16]	Progressive Deep Muscle Relaxation Training		Patients diagnosed with prostate cancer	Progressive deep muscle relaxation (n = 77) / Any intervention (n = 78)	Anxiety, depression and stress	Overall, there were significant changes over time in anxiety score and stress score between intervention and comparison groups. However, there was no significant change over time for depression score.	The improvement in anxiety and stress showed the potential of the intervention in the management of prostate cancer patients.
10	[27]	A psychosocial rehabilitation programme		Patients diagnosed with prostate cancer	Physical training (n = 53) / Information (n = 55) / Information plus physical training (n = 52) / Control (n = 51)	Depression, anxiety and quality of life	The control-group had comparatively ‘‘high”, and the physical training group ‘‘low”, mean value of depression at 12 months. Regarding the level of anxiety, all groups improved at the 12-month follow-up, but the Information group had a minor level of anxiety.	There is no synergetic effect of physical training and information as demonstrated by the PhysInfo values compared with only Phys or only Info, respectively.
								This study could not find any difference between psychosocial rehabilitation and no intervention.

HRQoL: Health Related Quality of Life; HEAC: Health education attention condition; CECT: Cognitive existential couple therapy; TIP-C: telephone interpersonal counselling

In the group interventions, all showed a reduction in depressive symptoms and in the couple interventions, there was a reduction in depressive symptoms and a favourable relationship cohesion. Those that did not have efficacy were based on mindfulness-based cognitive therapy [9], tele-based psycho-educational intervention [14] or the psychosocial rehabilitation program [25]. These studies also failed in providing cancer specific distress and quality of life.

Three studies provided information on the feasibility and acceptability of the intervention tested. The couple interventions were the most feasible and acceptable [10,11], followed by the technology-assisted psychosocial intervention [12]. Five studies concluded that future research was required to confirm the feasibility and acceptability and two did not provide a clear conclusion.

## 4. Discussion

A total of 15,094 records were collected from the literature search and 10 studies matched the inclusion and exclusion criteria for this systematic literature review. Of the 10 included interventions, nine interventions were for patients with prostate cancer and one for patients with kidney cancer—no interventions have been reported for BC to date. Depression was the most commonly reported endpoint and social, relationship and/or familial wellbeing were also well distributed through the majority of the studies. Three group interventions and two couple interventions showed positive efficacy outcomes. Couple interventions were observed to be the most feasible and acceptable.

The psychological implications and the significant decrease in health-related quality of life of the diagnosis and treatment of urological cancers have been extensively demonstrated [18]. This highlights the importance of providing supportive care particularly in terms of psychological and psychosocial support. Most studies to date were conducted in prostate cancer patients. Although prostate cancer is the most common urological cancer, it has been shown previously that other urological cancers, including BC [19], also have severe consequences for mental wellbeing of the patients—which may be related to slightly different treatment-related issues. A recent literature review specifically highlighted the prognostic implications of a mental illness on BC patients and how both the diagnosis and treatment of bladder cancer may affect mental wellbeing across various disease states [19]. Bladder cancer patients were also found to have an increased risk of suicide compared to prostate and kidney cancer [20]. In addition, a recent study showed that for kidney cancer, there is a significant number of patients with increased psychological distress and a consecutive need for psychosocial care [21]. A similar finding has been shown for patients with testicular or penile cancer [22]. Despite those reports, no study has been published evaluating a mental wellbeing intervention for patients with bladder, testicular or penile cancer in the last 10 years. However, for BC there was one recent systematic literature review which was focused on the effect of exercise to improve health-related outcomes in those patients undergoing radical cystectomy [23].

Even though no BC-specific interventions have been reported, intervention studies for other urological cancers published to date indicate that social support has an influence on the efficacy of the mental wellbeing intervention. Studies where a social or familiar support was available (group or couple interventions) were found to report better outcomes. In fact, in all group interventions studies, the positive efficacy was linked to the social support available [8,9,12]. For example, the expressive writing intervention was suggested to be beneficial for quality of life outcomes for patients who had social support available, including participants with depressive symptoms. In contrast, expressive writing may have suggested to not be beneficial or potentially even contraindicated for those lacking social support [11]. These findings align with the results of the study using a technology-assisted group-based psychosocial intervention [10]. Participation in the group consultation intervention showed to help men normalise their experiences and bolster hope, offsetting the increase in depressive symptoms reported by standard care participants. In addition, the technology-assisted psychosocial intervention resulted in meaningful differences for depressive symptoms and functional well-being. However, contrary to their hypothesis, the control group did report a better improvement in social wellbeing as compared to the intervention group—possibly due to the group dynamic of the control. More research, however, is needed to examine the interplay between social support and depressive symptoms for patients undergoing cancer treatment.

The studies that did not report a beneficial outcome of the intervention reported heterogeneity of the sample and small sample size as potential limitations. Furthermore, they highlighted the need for a sufficient number of sessions and an appropriate environment.

Feasibility of intervention studies focuses on testing procedures for their acceptability, estimating the likely rates of recruitment and retention of subjects, and the calculation of appropriate sample sizes. Evaluations are often undermined by problems of acceptability, compliance, delivery of the intervention, recruitment and retention [24]. The feasibility and acceptability of the included interventions were not clearly stated in most studies found. In addition, none of the interventions published to date included a component focused on patient acceptability. To allow for those interventions with positive efficacy to have an actual effect on mental wellbeing of patients with a urological cancer diagnosis, more emphasis should be put on feasibility and acceptability as to ensure an actual improvement in the patients’ experience [25].

## 5. Clinical implications and implications for further research

This literature review highlights the need for mental wellbeing interventions for BC patients and supports the hypothesis that group and/or couple interventions may be an acceptable approach to support patients and can potentially lead to a reduction in depressive symptoms and increase in relationship cohesion. While deeper understanding of feasible and acceptable interventions is needed, patients should be encouraged to seek support groups and couple therapy—an approach which needs to be assessed in more detail with respect to clinical efficacy and implementation in standard care.

## 6. Study limitations

This systematic review includes an extensive search through different databases, allowing for the inclusion of all types of interventions specifically focused on mental wellbeing. However, we were unable to provide summary statistics due to the heterogeneity in mental wellbeing parameters measured and variety in study design and interventions. This work provides the first step of a larger programme we are undertaking to improve the mental wellbeing of these patients—whilst following the MRC Framework for Development of Complex Interventions [24].

## 7. Conclusion

While awareness of the importance of mental wellbeing in BC patients is growing, this systematic literature review highlights the gap of feasible and acceptable interventions designed for these patients. Research into suitable mental wellbeing interventions is needed to help improve the experience of patients diagnosed with BC.

## Supporting information

S1 Appendix Protocol. (TIF)

S2 Appendix Search strategy. (TIF)

S3 Appendix Assessment of studies according to Down and Black checklist. (TIF)
