# Supplementary figures and images for: The need for supportive mental wellbeing interventions in bladder cancer patients: A systematic review of the literature
Source: PLoS One. 2021 Jan 28;16(1):e0243136. doi: 10.1371/journal.pone.0243136 (PMC7842965; doi:10.1371/journal.pone.0243136)

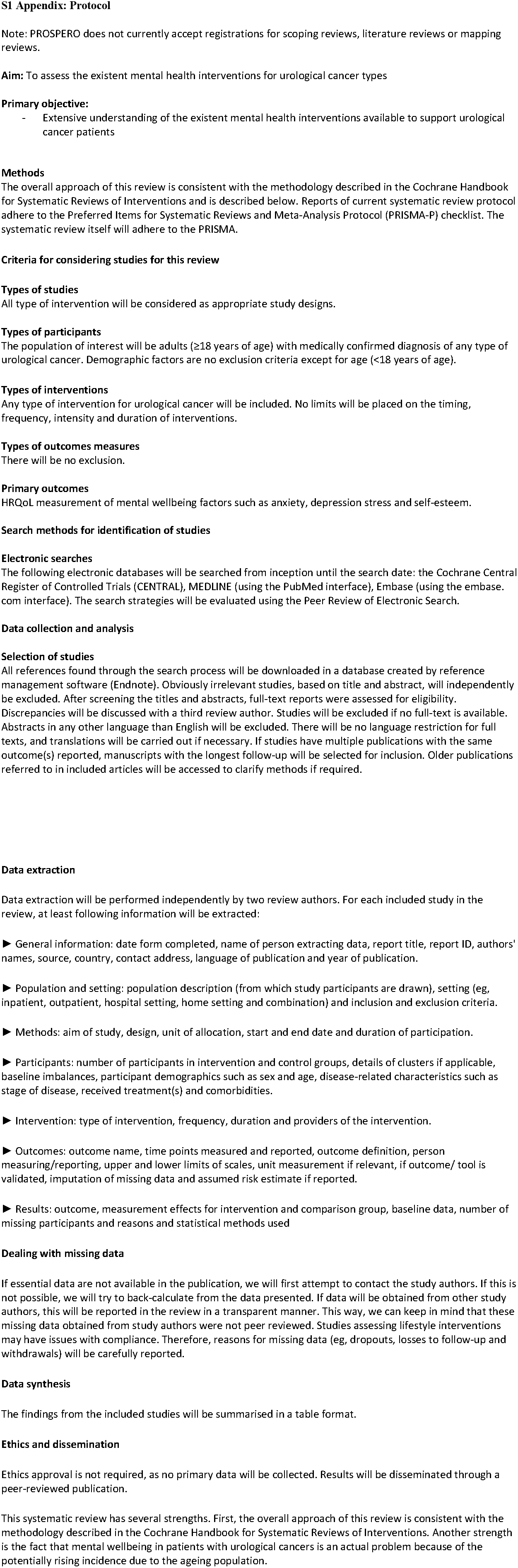

Supplement: S1 Appendix — (TIF) [file pone.0243136.s001.tif]

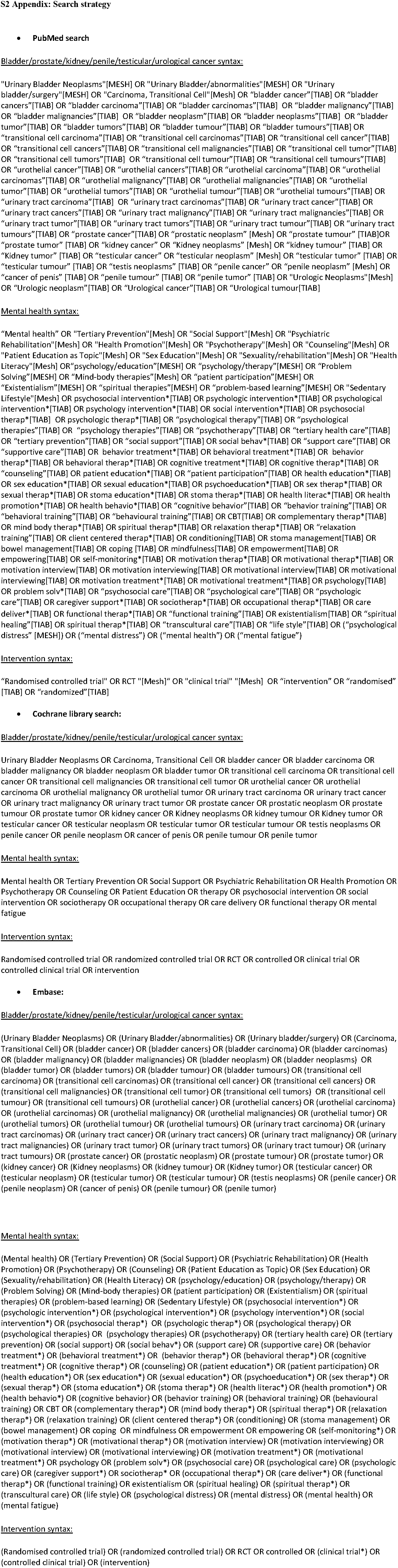

Supplement: S2 Appendix — (TIF) [file pone.0243136.s002.tif]

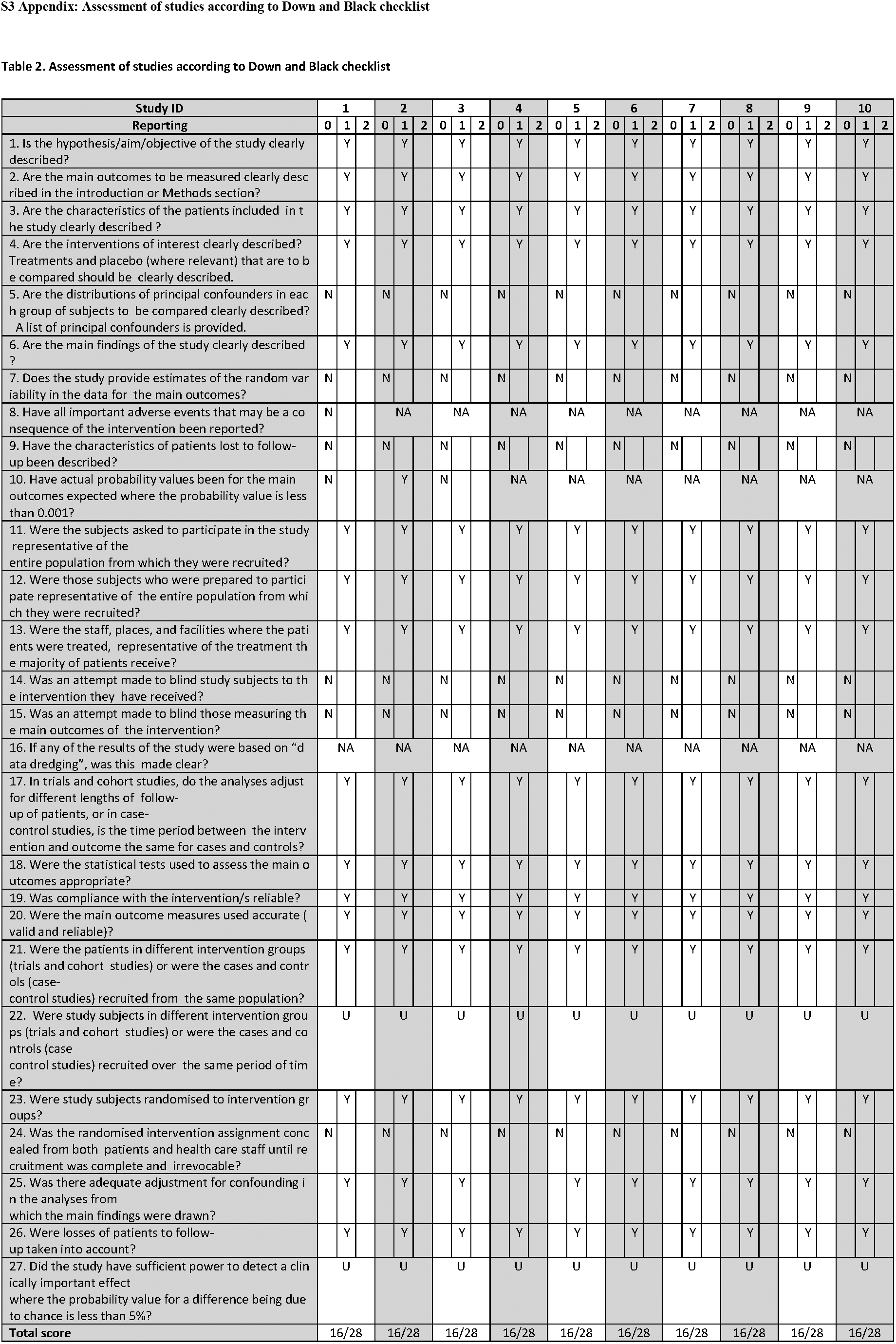

Supplement: S3 Appendix — (TIF) [file pone.0243136.s003.tif]
